# State rumination predicts inhibitory control failures and dysregulation of default, salience, and cognitive control networks in youth at risk of depressive relapse: Findings from the RuMeChange trial

**DOI:** 10.1016/j.jadr.2024.100729

**Published:** 2024-01-10

**Authors:** Henrietta Roberts, Mindy Westlund Schreiner, Stephanie Pocius, Alina K. Dillahunt, Brian Farstead, Daniel Feldman, Katie L. Bessette, Erin A. Kaufman, Will Slattery, Rachel H. Jacobs, David Jago, Sheila E. Crowell, Edward R Watkins, Scott A. Langenecker

**Affiliations:** a Mood Disorders Centre, School of Psychology, Sir Henry Wellcome Building for Mood Disorders Research, University of Exeter, Exeter EX4 4LN, UK; b University of Utah, USA; c University of Illinois at Chicago, USA; d Northwestern University - The Feinberg School of Medicine, USA; e University of California at Los Angeles, USA

**Keywords:** Rumination, Depression, Executive functions, Default-mode network, Cognitive control network, Adolescence

## Abstract

**Background:**

Trait rumination is a habitual response to negative experiences that can emerge during adolescence, increasing risk of depression. Trait rumination is correlated with poor inhibitory control (IC) and altered default mode network (DMN) and cognitive control network (CCN) engagement. Provoking state rumination in high ruminating youth permits investigation of rumination and IC at the neural level, highlighting potential treatment targets.

**Methods:**

Fifty-three high-ruminating youth were cued with an unresolved goal that provoked state rumination, then completed a modified Sustained Attention to Response Task (SART) that measures IC (commissions on no-go trials) in a functional MRI study. Thought probes measured state rumination about that unresolved goal and task-focused thoughts during the SART.

**Results:**

Greater state rumination during the SART was correlated with more IC failures. CCN engagement increased during rumination (relative to task-focus), including left dorsolateral prefrontal cortex and dorsalmedial prefrontal cortex. Relative to successful response suppression, DMN engagement increased during IC failures amongst individuals with higher state and trait rumination. Exploratory analyzes suggested more bothersome unresolved goals predicted higher left DLPFC activation during rumination.

**Limitations:**

The correlational research design did not permit a direct contrast of causal accounts of the relationship between rumination and IC.

**Conclusions:**

State rumination was associated with impaired IC and disrupted modulation of DMN and CCN. Increased CCN engagement during rumination suggested effortful suppression of negative thoughts, and this was greater for more bothersome unresolved goals. Relative task disengagement was observed during rumination-related errors. DMN-CCN dysregulation in high-ruminating youth may be an important treatment target.

## 1. Introduction

Trait depressive rumination is a style of negative repetitive thinking that can emerge during adolescence and constitutes a major risk factor for psychopathology (Ehring and Watkins, 2008; Nolen-Hoeksema, 1991, 2000; Nolen-Hoeksema et al., 2008, Watkins, 2008; Watkins and Roberts, 2020). Trait rumination involves passive, repetitive focus on personal difficulties, negative mood, and unresolved goals (Martin and Tesser, 1996; Moberly and Watkins, 2008; Roberts et al., 2013; Watkins, 2008; Watkins and Roberts, 2020), and is conceptualized as an overlearned cognitive habit that disrupts the contextual sensitivity and temporal specificity of problem-solving, instead worsening mood (Carver, 1996; Watkins, 2008; Watkins and Nolen-Hoeksema, 2014; Watkins and Roberts, 2020). There is a distinction between the depressive ruminative style (*trait rumination*: a chronic maladaptive thinking style) and discrete ruminative periods (*state rumination:* provoked by unresolved goals or stressors and not necessarily problematic). Experimental research demonstrates that unsatisfactory goal progress causes *state* rumination, and individuals with high *trait* rumination are more susceptible to episodes of state rumination, with these episodes becoming longer, more intense, and more disruptive (Roberts et al., 2013, 2020). Whilst there is a substantial literature examining the negative consequences of trait depressive rumination, relatively fewer studies have examined the interaction between state rumination about unresolved problems and concurrent functioning as rumination occurs. This is important for understanding how responses to active problems impact on cognitive functioning and emotion regulation and has implications for intervening to prevent or disrupt the emergence of the depressive ruminative response style.

Trait rumination is associated with impaired performance on tasks that index executive functions (EFs; Joormann et al. 2007; Koster et al. 2011; Roberts et al. 2017; Watkins and Roberts 2020; Zetsche et al. 2018). EFs support goal-directed behavior and enable individuals to successfully modulate attention and over-ride habitual, prepotent, or automatic processes. Studies demonstrate that higher trait rumination is correlated with poorer updating of working memory (Joormann and Gotlib, 2008), task-switching (De Lissnyder et al., 2012) and inhibitory control (Shimony et al., 2021; Whitmer and Banich, 2007). EF deficits may therefore be particularly problematic for individuals with high trait rumination by disrupting the ability to overcome spontaneous ruminations and sustain task-focus (Joormann et al., 2007; Koster et al., 2011). A complementary hypothesis is that state rumination reduces available cognitive resources, thereby impairing concurrent performance on EF tasks (Ellis and Ashbrook, 1988; Hartlage et al., 1993; Hertel, 2004; Watkins and Brown, 2002). The balance of evidence suggests that the relationship between rumination and EFs is reciprocal: EF deficits increase susceptibility to rumination, and rumination further interferes with the application of EFs (Hartlage et al., 1993; Joormann and Gotlib, 2010; Koster et al., 2011; Roberts et al., 2017; Watkins and Roberts, 2020).

Rumination is linked to abnormal functioning in brain networks that are implicated in the modulation of cognitive control, self-referential processing, and emotion perception, processing, and regulation (Fox et al., 2018; Hamilton et al., 2015; Watkins and Roberts, 2020). There is evidence of increased activation of regions within the default mode network (DMN), extending into the subgenual prefrontal cortex (sgPFC), during state rumination (Burkehouse et al., 2017; Chen and Yan, 2021; Cooney et al., 2010; Hamilton et al., 2015; Nejad et al., 2013). Likewise, resting state fMRI studies report that rumination is associated with disrupted and elevated connectivity within the DMN, and elevated connectivity between DMN and cognitive control (CCN) and salience and emotion networks (SEN) (Berman et al., 2011; Lois and Wessa, 2016; Zhou et al., 2020). The DMN supports self-referential processing and external task-independent (‘task negative’) thought and includes the posterior cingulate cortex (PCC) and medial prefrontal cortex (mPFC) (Christoff et al., 2009; Fox et al., 2018; Raichle, 2015; Zhou et al., 2020). The CCN, which supports EFs, including inhibitory control, shows increased activation within the dorsolateral prefrontal cortex (DLPFC) during both state rumination and inhibition of responses to affective material among high trait ruminators (Cooney et al., 2010; Vanderhasselt et al., 2011). The left DLPFC is particularly implicated in inhibiting negative affective stimuli and regulating negative representations among depressed patients (Holmes and Pizzagalli, 2008; Leyman et al., 2009, 2011; Vanderhasselt et al., 2009; Marchetti et al., 2012). Therefore, rumination may be linked to inhibitory control via failure to regulate or switch away from self-referential negative content (Joormann et al., 2007; Koster et al., 2011; Marchetti et al., 2012; Nejad et al., 2013; Shimony et al., 2021; Watkins and Roberts, 2020).

Rumination is also correlated with SEN engagement (Burkehouse et al., 2017; Mandell et al., 2014; Nejad et al., 2013; Ray et al., 2005;Siegle et al., 2002), which detects and integrates salient emotional information. Trait rumination has been linked to emotional salience regions including the amygdala, which is implicated in automatic attention to negative information in depression, and the anterior insula, hippocampus, and PCC, which have been implicated in elaborative processing of negative emotional content (Mandell et al., 2014). Burkehouse et al. (2017) found that relative to healthy controls, youth with remitted depression exhibited elevated activation in emotion processing regions including the amygdala and insula during a rumination induction task, suggesting that the link between emotional salience regions and rumination persists during remission from a depressive episode. Atypical interactions between the CCN, DMN, and SEN may constitute key treatment targets that underlie the persistence of depressive rumination (Nejad et al., 2013). The persistence of DMN engagement during the transition from rest-based to externally-focused tasks predicts attentional lapses and task errors (Marchetti et al., 2012; Prado and Weissman, 2011; Sonuga-Barke and Castellanos, 2007); however, this has not been examined in the context of rumination. Individuals with trait rumination exhibit increased CCN engagement during cognitive tasks with affective stimuli, as well as relative DMN dominance over CCN during rest. This is hypothesised to reflect the disruptive effect of rumination on cognitive function (Berman et al., 2007; Hamilton et al., 2011; Marchetti et al., 2012; Vanderhasselt et al., 2011).

To date, few studies have examined the relations between uninstructed state rumination during a concurrent EF task and task performance at the neural level. Fewer still have studied these relations in youth with depression history and high trait rumination, where these processes are relatively less confounded by the complex illness and treatment histories that can present in adult depression. Examining uninstructed provoked rumination about idiographic unresolved goals is likely to be more ecologically valid and temporally sensitive than using an instructed induction task or questionnaire. Behavioral tasks that capture how rumination interferes with performance *as it occurs* are essential for investigating proximal neural mechanisms of maladaptive state rumination. Importantly, this design permits us to examine relations between occurrences of state rumination, relative levels of trait rumination, behavioral lapses in EF, and DMN, SEN and CCN engagement during both state rumination and execution of inhibitory control. For individuals higher in trait depressive rumination, periods of state rumination are expected to be more frequent, intense, negative, and disruptive. At the neural level, rumination -related errors would be expected to be associated with greater recruitment of emotional salience regions for individuals higher in trait rumination who are reporting more frequent ruminative intrusions (i.e., greater state rumination) during the EF task. We therefore predicted that the greater the tendency to depressive rumination, the more negative and salient state ruminative content would be during rumination-induced IC-RS failures. Such mechanistic provocation studies rely on oversampling those with high trait disposition to experience state rumination, offering the compelling prospect of identifying proximal targets for neural modulation in real time.

The modified Sustained Attention to Response Task (SART) is a go/no-go EF paradigm that includes pseudo-randomly presented thought probes to measure occurrences of state rumination about an unresolved personal goal (Roberts et al., 2013), as compared to task focus, or other thoughts. Lapses in EF during the SART are indexed by faster reaction times (RTs) on go-trials (targets) and more commission errors on no-go (lure) trials. Responses capture the strength of inhibitory control response suppression (IC-RS) (Carter et al., 2013; Grahn and Manly, 2012; McVay and Kane, 2009; Robertson et al., 1997; Stevenson et al., 2011). There is evidence that off-task thinking during SART is temporally related to commission errors whereby individuals make significantly more commission errors during periods before off-task thought than before task-focused thought (Christoff et al., 2009) and are more likely to be engaged in off-task-thinking in the period immediately before an error (Robertson et al., 1997; Smallwood et l., 2004). IC-RS is a key aspect of inhibitory control that emerges in late childhood and continues to mature over the course of middle and late adolescence (Bessette et al., 2020). Adolescence therefore offers a prime window of opportunity for studying neural mechanisms underlying pathological rumination and identifying targets for early intervention.

The present study uses the SART to examine associations between behavioral and neural indices of rumination and EF among youth with remitted depression and elevated trait rumination. We hypothesized that greater state rumination during the SART would predict (1) more commission errors on lure trials and (2) faster reaction times to targets. We further hypothesized (3) higher trait rumination, (4) state rumination during the SART, and (5) their interaction, would predict increased SEN activation during commission errors. At an exploratory level, we predicted (6) thought epochs about the unresolved goal (state rumination) would exhibit increased activation within DMN and decreased activation in CCN as compared to thought epochs focused on the task.

## 2. Methods and materials

### 2.1. Participants and procedure

Participants were recruited from University of Utah hospitals, clinics, and via local advertising on Facebook, Instagram, online advertisements and radio stations as part of a clinical trial protocol evaluating rumination focused cognitive behavior therapy (see Roberts et al., 2021 for full trial protocol) with a total of 60 participants required for the trial to have power of .99 to detect an effect of therapy of .5 standard deviation (SD, effect) size change, and with known reliability of RRS (*r* = .77) and power of .98 to detect an effect on resting state functional magnetic resonance imaging (rs-fMRI) connectivity of left Posterior Cingulate Cortex (PCC) to right inferior frontal gyrus (IFG) (*r* =.71).

Participants were postpubertal (assessed using the Peterson Pubertal Development Scale; Peterson et al. 1988) adolescents aged 14-17 with a previous diagnosis of Major Depressive Disorder (MDD), based on Diagnostic and Statistical Manual (DSM-5; American Psychological Association 2013) criteria and confirmed using a comprehensive clinician-administered diagnostic assessment including the Kiddie Schedule for Affective Disorders and Schizophrenia – Present and Lifetime Version DSM-5 (KSADS-PL; Kaufman et al. 2016), which screened for suicidality. Trait Rumination was assessed using the Ruminative Responses Scale of the Response Styles Questionnaire (RRS; Treynor et al. 2003), a 22-item measure, with higher total scores representing a greater trait tendency toward rumination (range: 22–88). The RRS has high internal consistency, acceptable construct validity, and good test-retest reliability (Nolen-Hoeksema and Morrow, 1991; Treynor et al., 2003). An age-adjusted total cut-off score from 28 to 31 for males and 35–38 for females was used as age increased from 14 to 17. This strategy approximates a T-score above average (> 50; Jose and Brown 2008). We therefore selected youth with elevated depressive rumination, whilst allowing for a range of levels of severity in accordance with RDoC (the range in our sample was 30–80). The RRS includes two 5-item subscales that measure brooding and reflection. Brooding is conceptualised as negative and evaluative focus on the self and is argued to be the most unhelpful form of rumination; reflection is conceptualised as a purposeful focus on problem solving and is argued to be less problematic. Both RRS total scores, and scores on the brooding subscale were calculated for all participants. Due to our primary focus on the impact of rumination, residual depression and anxiety symptoms were assessed as confounds of non-interest using the Children’s Depression Rating Scale – Revised (CDRS-R; Poznanski et al. 1996; validated for use in adolescents by Mayes et al. (2010) total score, the Reynolds Adolescent Depression Scale Short-Form (RADS-SF; Reynolds 2008) total score, and the Screen for Child Anxiety Related Disorders (SCARED; Birmaher et al. 1997) generalized anxiety score. The RADS-SF has acceptable reliability and validity (Milfont et al., 2008), the SCARED has strong test-retest reliability, and adequate external validity with a clinician-rated measure of anxiety (Behrens et al., 2019).

Key exclusion criteria included lifetime history of conduct disorder, autism spectrum disorder, any psychotic disorder, or bipolar disorder; an estimated intelligence quotient (IQ) of 75 or less as measured by the Weschler Abbreviated Scale of Intelligence (WASI); current clinically significant depressive symptoms indicated by a raw score greater than 45 on the CDRS-R; and currently endorsed suicide attempt or plan within the past six months (see NCT03859297 for additional exclusions).

Informed consent and assent were obtained from participants and a legal guardian before completion of the diagnostic interview and the diagnostic questionnaires. The mean time between completion of the diagnostic interview and the fMRI task was 21 days. This study was reviewed and approved by the University of Utah Institutional Review Board and the University of Exeter Psychology Ethics Committee.

### 2.2. MRI

#### 2.2.1. Unresolved goal SART

**Fig. 1.**
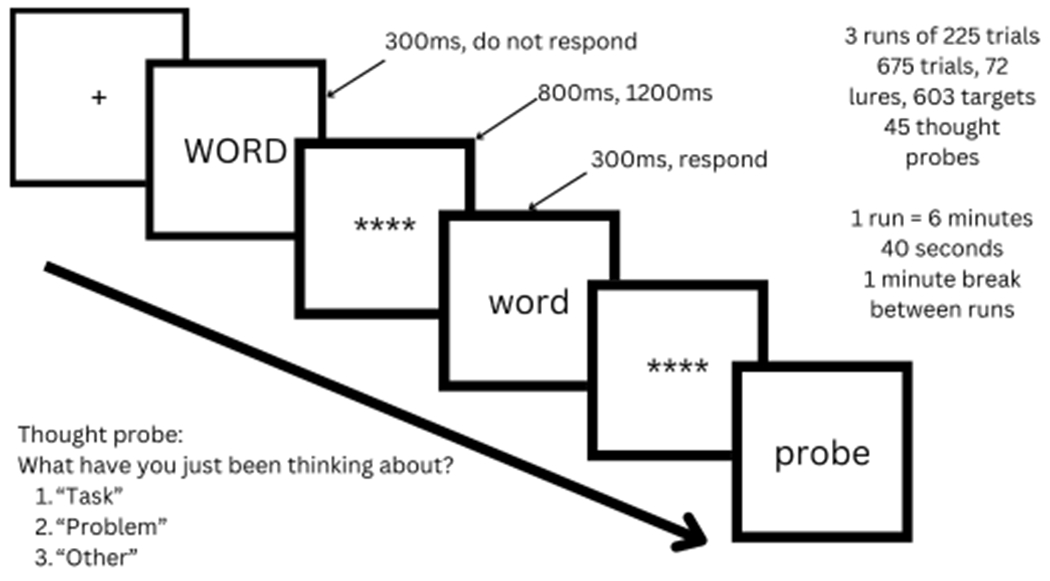
The modified Sustained Attention to Response Task (SART). Following a fixation cross, participants viewed a word for 300 ms and then a jittered mask (800 ms, 1200 ms). Participants were instructed to respond to words in lower case but withhold their response for upper case words.

The fMRI task (Fig. 1) comprised three six-minute runs of the modified Sustained Attention to Response Task (SART; adapted from 49). Prior to entering the scanner, participants were asked to generate a current unresolved goal that had been repeatedly coming into their mind and bothering them during the past week, to provoke rumination (see Roberts et al. 2013 for detailed description of the goal cueing procedure). For 12 participants, this took place on the day of scanning; due to Covid-19 the remaining participants completed this task by video conference 1-3 days before the scan. Participants briefly described the problem and rated it from 1 (very little) to 9 (very much) for: (1) importance, (2) bothering now, (3) bothered at worst, (4) number of thoughts about unresolved goal during past week, (5) problem duration (weeks). Problem descriptions and ratings were screened to confirm that an appropriate problem had been generated and rating of problem importance, current bothersomeness, and thoughts in the past week were 4 or above. Common themes among the unresolved goals that our sample identified were relational difficulties, bereavement, difficulties at school, and difficulties at home. Prior to commencing the SART, an experimenter confirmed with participants that their problem remained unresolved. At the start of each run of the SART, participants viewed a 40s prompt that instructed them to focus on that unresolved goal. It was then explained that participants would next view a continuous string of words and they were instructed to make a button press response to each word presented in lowercase (targets) and withhold their response for words presented in uppercase (lures). Participants were instructed to respond to the words as quickly as possible while still maintaining their accuracy. Stimuli were taken from the perceptual SART reported in McVay and Kane (2009) (e.g., sunflower, football, spatula), and were identical to those used in Roberts et al. (2013). A fixation cross was presented before each word was presented for 300 ms followed by a jittered mask (800–1200 ms). Thought probes were presented pseudo-randomly throughout the task, asking participants to indicate the focus of their attention immediately prior to the probe.

Key SART dependent variables were:

1. Rumination: percentage of thought probes for which participants endorsed focusing on their unresolved goal (PercentRum); mean number of consecutive probes focused on the unresolved goal (MeanRumDuration); maximum number of consecutive probes focused on the unresolved goal (MaxRumDuration).
2. IC-RS: Errors of commission (Commissions: failure to withhold response on a lure trial, measures IC-RS), mean RT to targets (faster RTs to targets are associated with EF lapses), SD of target RTs (reaction time variability, higher variability is associated with more EF lapses).

#### 2.2.2. fMRI acquisition preprocessing and analysis

Imaging data was collected using a 3T Siemens Prisma scanner that included acquisition of axial oblique images using multiband (MB = 6). Parameters included TR (repetition time) = 800 ms, FOV (field of view) = 216 mm, 2.4 mm isotropic voxels, TE (echo time) = 30, and a flip angle of 52°. We acquired two fieldmaps in opposite phase encoding directions for distortion correction.

Neuroimage preprocessing included removing the first 10 volumes to reduce saturation effects. Preprocessing included use of ANIMA (https://anima.irisa.fr/) and Statistical Parametric Mapping 12 (SPM12) software packages and consisted of 7 steps: echo-planar imaging (EPI) distortion correction (ANIMA), realignment of time-series data (SPM12), co-registration of high-resolution T1 to time-series (SPM12), tissue segmentation of high-resolution T1 images (SPM12), normalization of high-resolution images to MNI space (SPM12), normalization of functional images to MNI space based on high-resolution MNI space output (SPM12), and application of Gaussian smoothing using a 5 mm kernel (SPM12).

The first model was an event-related task design, coding correct responses for targets (lower case words) and incorrect responses for commissions (upper case words).

We also included a second exploratory model to examine momentary shifts in state rumination (to better align with performance). Since we could not acquire a firm onset or offset time for when a participant would be thinking of the unresolved goal versus the task, we examined blocks of time 4 s before, and eight seconds after the thought probe response (e.g., proximal, “fuzzy” block design). This 12 s epoch is likely a cleaner, more stable, and less noisy approximation of the current mental state as a block (of time), and comparative data driven approaches have many challenges (Jabakhanji et al., 2022): when predicting a mental process from voxel responses (as opposed to modelling brain activation patterns caused by a mental process), the number of voxels is typically much larger than the number of observations, which leads to an imprecise problem with potentially infinite solutions. Thought probes were presented 15 times per run, at pseudorandom intervals independent from no-go trials. Both the event-related and thought probe block designs included six realignment estimates of movement.

We created regressors for the Commissions – Targets contrast (activation during Commission errors relative to correct Targets) and added regressors of interest: PercentRum (state), RRS score (trait), and the interaction term of these two (statextrait_Rumination). We also included regressors of non-interest (SCARED generalized anxiety score (in our data SCARED generalized anxiety score was correlated with both RRS total and RRS brooding, which was not the case for SCARED total), CDRS, summary movement deviation factor, and confidence estimate for number of commission trials). The summary movement deviation factor is derived from calculating the standard deviation of movements in roll, pitch, and yaw across all three runs, and then subjecting those values to a principal components analysis (which was significantly correlated with each movement deviation value). We used a cluster-level correction combined p-value of .005 and cluster size of 96 contiguous voxels (adjusted *p*-value of *p* < .05).

Thought probe (fuzzy block) brain imaging analyzes were block design analyzes of 45 thought probes spread across the three runs. To be included in these exploratory second level models, individuals needed to have: (1) valid performance data (i.e., no technical errors with recording their performance data), (2) at least one correct rejection, (3) at least one thought probe (TP) self-described as focusing on the task, and (4) at least one thought probe self-described as focusing on the unresolved goal. The primary contrast of interest was TP_Problem (focus on the unresolved goal) minus TP_Task (focus on task). Regressors of interest and non-interest were the same as above. A cluster level correction combined p-value of .005 and cluster extent of 81 contiguous voxels was FWE level significant at *p* < .05 at whole brain level for these analyzes.

### 2.3. Analytic plan

Bivariate correlations tested the hypotheses that greater state rumination during the SART would predict (1) more commission errors on lure trials and (2) faster reaction times to targets. Regression models were constructed in matlab/SPM 12 to test the prediction that (3) higher trait rumination (RRS total), (4) state rumination during the SART (percentage rumination on the thought probes), and (5) their interaction (RRS total x PercentRum), would predict increased SEN activation during commission errors. To do this, first level contrasts (Commission errors – correct targets) were constructed for each individual and entered into a second level regression model along with RRS total and PercentRum and their interaction. At an exploratory level, a regression model examined in matlab/SPM 12 whether increased activation within DMN and decreased activation in CCN was observed during thought epochs about the unresolved goal (state rumination) as compared to activation during thought epochs focused on the task. First level contrasts (Problem responses – task response on the thought probes) were constructed and entered into a second level regression model along with RRS total and PercentRum. Exploratory analyzes were the conducted to further investigate activation derived from the TP_Problem-TP_Task contrast. We extracted activation from the seven regions observed in the regression model, using MARSBAR (MARSeille Boîte À Région d’Intérêt toolbox for SPM). We then entered these into a regression model in SPSS along with participant ratings of how much their unresolved goal (1) bothered them now, and (2) bothered them at its worst. Current salience and “bothersomeness” of the unresolved problem varied from 1 (very little) to 9 (very much). This analysis allowed us to explore the possibility that the emotional salience and bothersomeness of current problems facilitates neural responses to rumination.

## 3. Results

### 3.1. Participant characteristics

Participants’ demographic and clinical characteristics are summarized in Table 1. A CONSORT diagram reporting participant flow throughout the wider clinical trial in which the study was embedded is published in the main trial outcomes paper with treatment change and resting state fMRI (Langenecker et al., 2024). 114 youth completed the initial diagnostic assessment, of whom fifty-three were entered into the main analyzes. Additional youth completed the MRI task but are not included in any analyzes (*n* = 11) due to not having the minimum data required to analyze the thought probe and SART performance data: Five participants reported no task-related thoughts during thought probes, one participant reported no ruminative thoughts, four participants had a 100% error rate, one participant was missing accuracy data due to a data-recording error.

### 3.2. State rumination and attentional lapses during the SART

SART thought probe responses and performance indices are summarized in Table 1. As predicted, all three indices of state rumination during the SART were correlated with more commission errors: PercentRum (*r* (53) = .44, *p* = .001), MeanDurRum (*r* (53) = .41, *p* = .002), MaxDurRum (*r* (53) = .39, *p* = .004). There were no significant associations between state rumination measures and omission errors (failure to respond to a target; all *p*s > .080). State rumination was also correlated with faster RTs to targets: PercentRum (*r* (53) = −.41, *p* = .002), MeanDurRum (*r* (53) = −.34, *p* = .01), MaxDurRum (*r* (53) = −.32, *p* = .02). There were no significant associations with RT variability (all *p*s > .16). There were no significant associations between percentage of task-focused thoughts during thought probes, and SART performance (all *p*s > .35). Thoughts about ‘other’ topics were correlated with fewer commission errors (*r* (53) = −.39, *p* = .004) and slower RTs to targets (*r* (53) = .38, *p* = .005), indicating a more conservative response style.

Due to experimenter error, one participant who rated “thoughts past week” as 3, and four participants who rated “bothers now” below 4 proceeded with these goals during the goal cueing task and fMRI task. To determine whether the unresolved goal task had successfully provoked state rumination for these participants, we examined the percentage of thought probes for which they reported thinking about their unresolved goal. These were 64.44% probes, 6.67% probes, 66.67% probes, 22.22% probes, and 33.33 % probes respectively, suggesting that these participants were experiencing thoughts about the unresolved problem during the SART across a similar range of values to other participants (2.22–64.44% in the rest of the sample). Repeating the behavioral analyzes excluding (a) all of these participants, and (b) the one participant reporting relatively lower state rumination during the SART (rumination on 6.67% of probes) did not qualitatively alter the results. The exception was of one significant positive correlation between average length of rumination and RT variability during the SART when all these participants (of questionable validity) were excluded, *r* (48) = .384, *p* = 0.007.^1^

### 3.3. Trait rumination and state rumination during the SART

Trait rumination (RRS total, and brooding subscale) was not correlated with reported indices of state rumination during the SART (all *p*s > .16), nor were they correlated with measures of SART performance (all *p*s > .10). This may be due to our selecting a sample of youth with a high propensity to ruminate (resulting in reduced range of RRS scores); moreover there is no expectation of a linear relationship between a general trait propensity and an experimental manipulation.

### 3.4. Neural activation during errors of commission relative to correct target (go-trial) responses (commissions-targets)

**Fig. 2.**
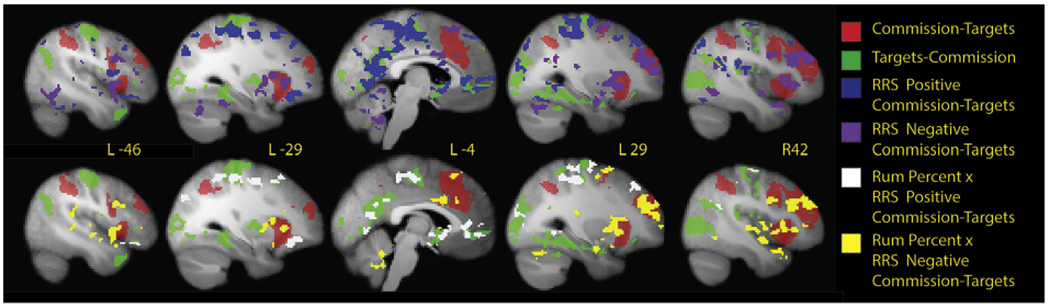
Activation during errors of commission (relative to correct target responses, Commissions-Targets) in red. The inverse contrast of Targets-Commissions is in green. Dimensional relationships of the Commissions-Targets activation with RRS (blue = positive relationships and purple = negative relationships), as well as the positive (white increasing) and negative (yellow decreasing) interaction of RRS and PercentRum relationships with Commissions-Targets Activation.

As expected, the Commissions – Targets contrast highlighted prominent activation in bilateral dorsal and lateral frontal regions associated with error detection (aligned with SEN): left anterior insula and bilateral dorsolateral PFC (DLPFC) (Fig. 2 illustrates some key regions; detailed information about the individual brain areas encompassed within these broader brain regions can be found in Supplemental Table 1). For the Commissions – Targets contrast there was increased activation in relation to higher RRS scores (RRS positive) in bilateral motor and somatosensory cortices, left anterior cingulate cortex (ACC) and ventral striatum, and PCC. There was decreased activation for Commissions - Targets in relation to higher RRS scores (RRS negative) in bilateral DLPFC, anterior insula, inferior and middle frontal gyrus, lateral posterior temporal cortex, and right caudate. The interaction term was significant for nearly the same bilateral regions as those associated with RRS positive (increasing) and negative (inverted; Fig. 2). The Target – Commission contrast activation pattern included left-lateralized primary and secondary motor cortex, and areas within bilateral DMN including SGC and PCC, and posterior ventral temporal regions.

### 3.5. Unresolved goal responses to the thought probes (state rumination probes)

**Fig. 3.**
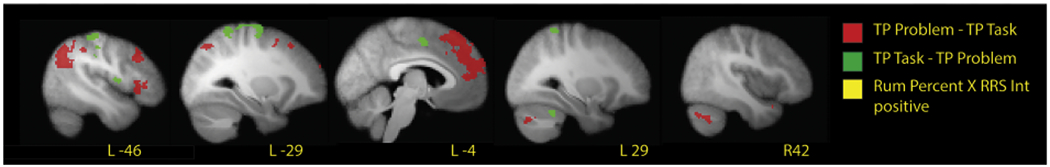
Activation during unresolved goal responses to the thought probes (relative to task-focused responses, TP_Unresolved Goal minus TP Task). This TP_Unresolved Goal minus TP Task contrast is shown in red, and the inverse is in green. The Percent Rumination x RRS positive interaction is shown in yellow.

The TP_Problem – TP_Task contrast revealed activation in dorsomedial DMN extending spatially into SEN (Fig. 3; information about the individual brain areas encompassed within these broader brain regions can be found in Table 2), including Dorsomedial PFC (DMPFC, BA 8 and 9) and right temporal pole. Specifically, a pattern of ventral and left DLPFC and inferior parietal lobule activation emerged that crossed over in junction points of the CCN and DMN. There was little evidence of activation within the ventral-medial DMN. For the TP_Task – TP_Problem there was increased activation in the superior parietal lobule (BA 7), left insula, and left ventral ACC. There was increased activation for TP_Problem - TP_Task in relation to higher PercentRum in the temporal-parietal junction bilaterally, and this same pattern was evident in the negative RRS interaction contrast.

**Fig. 4.**
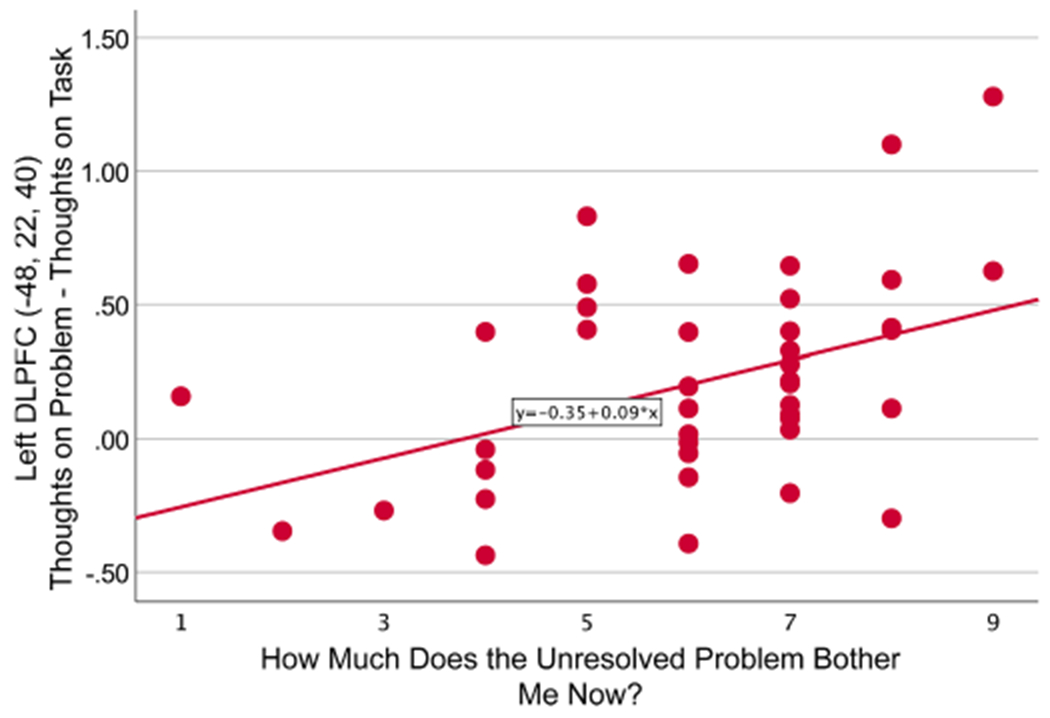
Interaction between Left DLPFC activation in Problem-Task contrast and ratings of current bothersomeness of unresolved goal.

Exploratory analyzes of the relationship between emotional salience of ruminative problems and neural activation during rumination revealed that three of the seven ROIs (bilateral dorsal ACC *r* = .35, *p* =.02, left DLPFC *r* = .42, *p* = .005 (Fig. 4), and left inferior parietal lobule *r* = .34, *p* = .03) from the TP_Problem-TP_Task contrast showed a significant correlation between current bothersomeness and degree of activation difference in this contrast (and all correlations of activation with bothersomeness were positive *r*s > .16). Those with most active state rumination for the unresolved problem observed the greatest degree of difference in activation with core bilateral error detection (SEN) regions when thinking about the unresolved problem relative to the task. Peak bothersomeness (*B* =−.16, *p* =.31) and current bothersomeness (*B* = .48, *p*=.003), predicted activation differences for left DLPFC TP_Problem-TP_Task, suggesting that current bothersomeness and high trait rumination facilitate increased activation in many of these areas. Due to the exploratory nature of these analyzes, we examined whether these findings remained reliable when a Bonferroni adjustment was applied, after which only the correlation between current bothersomeness and degree of activation remained significant for left DLPFC.

## 4. Discussion

We hypothesized that greater state rumination during the SART (measured using thought probes) would predict (1) more errors of commission, and (2) faster RTs. Both hypotheses were supported, suggesting impaired application of EF in these high state rumination periods. At the neural level, we hypothesized that (3) higher trait rumination, (4) state rumination during the SART, and (5) their interaction, would predict increased SEN activation during commission errors. Higher state and trait rumination interacted to predict greater SEN and DMN engagement during errors, consistent with a pattern of task disengagement and self-reflective distraction. We also predicted (6) that there would be increased DMN and decreased CCN activation during thought epochs about the unresolved goal (state rumination), relative to thought epochs about the task. However, there was little evidence that state rumination was associated with increased activation in ventromedial or posterior cingulate areas within the DMN. Activation was observed in a borderline region of dorsomedial frontal cingulate, where the DMN converges with the SEN. During state rumination (relative to task), there was a focus of activation for a left lateralized network of frontal and parietal regions (SEN), more so with higher levels of state rumination and current bothersomeness of the unresolved goal. This pattern highlighted increased left DLPFC activation during state rumination in the SART, relative to task-focused thoughts, which is consistent with previous evidence that induced rumination is associated with enhanced engagement of left DLPFC in depression (Cooney et al., 2010; Vanderhasselt et al., 2011). This may reflect attempts to overcome interference between rumination and task (e.g., dual-tasking) or effortful problem-solving.

Notably, DLPFC has been implicated in the inhibition and regulation of negative affective stimuli and rest-to-affective-task transitions in patients with depression (Holmes and Pizzagalli, 2008; Leyman et al., 2009, 2011; Marchetti et al., 2012; Vanderhasselt et al., 2009). Therefore, one interpretation of these findings is that individuals prone to rumination exhibit greater CCN recruitment as a compensatory response attempting to overcome emotionally salient and ruminative content (Marchetti et al., 2012; Vanderhasselt et al., 2011). Regions associated with conflict resolution and errors were also engaged, particularly in DMPFC (part of the SEN; Fig. 3). There may be an abnormal overlap between task positive and task negative network engagement in depression (Marchetti et al., 2012), with the DMPFC (the ‘dorsal nexus’) identified as the key region that distinguished depressed and never depressed individuals [66]. These unexpected findings are consistent with the hypothesis that DMPFC dysregulation may underlie depression-related EF impairments, as well as increased automatic responding and negative self-focused attention (rumination; Bessette et al. 2020; Sheline et al. 2010).

This study provides compelling evidence of the neural correlates of uninstructed provoked state rumination about unresolved personal goals in youth. Despite considerable theoretical elaboration, relatively little empirical research has investigated the interplay between trait rumination and episodes of state rumination about unresolved goals, and the neural mechanisms underlying this relationship remain to be elucidated. Our findings suggest that neural activation during state rumination about unresolved goals and activation correlated with trait depressive rumination may not be synonymous. Consistent with previous studies, there was evidence that state rumination was associated with increased engagement of the DLPFC. This suggests that while trait depressive ruminators exhibit a pattern of increased DMN engagement during rest, regions of the CCN and SEN become relatively engaged during episodes of state rumination. It is possible that state rumination during the SART reflects a more active process, consistent with repeated attempts at problem-solving, as opposed to the more passive dwelling on negative feelings that is typically described in trait depressive rumination. Alternatively, individuals reporting episodes of state rumination during the SART may be engaging in a process of active suppression and increased cognitive engagement to try to overcome negative ruminations and restore task-focused attention. State rumination was additionally associated with increased engagement of the DMPFC, lending further support to the hypothesis that DMPFC dysregulation in depression may facilitate increased negative self-referential thinking, as well as impaired attentional control.

Increased DMN engagement during errors of commission among individuals with higher levels of trait rumination and frequent state rumination during the task may suggest a pattern of task disengagement and internal self-focus. It has been argued that DMN disruptions can be defined as a ‘depressive scar’ (Lewinsohn et al., 1981; Marchetti et al., 2012; Sheline et al., 2010), which manifest in dysregulated switching between internally and externally-oriented attention, leading to increased rumination and impaired inhibitory control (Joormann et al., 2007; Koster et al., 2011; Marchetti et al., 2012; Nejad et al., 2013). Indeed, previous studies demonstrate that the persistence of DMN engagement during the transition from rest-to-task predicts attentional lapses and task errors (Prado and Weissman, 2011; Sonuga-Barke and Castellanos, 2007), but the hypothesised link with rumination was not systematically examined prior to this report. Our findings suggest this may be a promising avenue for further work.

Our behavioral data indicate that more frequent and persistent state rumination was associated with more commission errors and faster RTs, suggesting weakened, surface application of EF. These findings can be interpreted as evidence that episodes of state rumination disrupt concurrent application of EF to SART performance via weakened IC-RS. The observed increase in DMN engagement during errors for high ruminators is also consistent with this interpretation.

Study limitations include the correlational nature of the research design, which did not permit a direct contrast of the causal accounts of the relationship between state rumination and EF impairments or examine the developmental trajectory of rumination longitudinally. Studies using cognitive training or neural modulation, as well as contrasting state rumination with a non-ruminative control condition, would permit the development of more sophisticated causal models at the levels of both brain and behavior. While our sample had high trait rumination, there was still considerable variability (aligned with a Research Domain Criteria, RDoC, framework; RRS range 30-80 in our sample). The interaction between state and trait rumination during errors suggests that a useful next step will be to evaluate these models in samples with relatively clinically severe (e.g., >60) and low-risk (e.g., <35) trait rumination, to determine if this pattern is more pronounced in those with high levels of vulnerability. Our exploratory results suggest that, in the context of ruminative vulnerability (trait), a current problem trigger requires a sufficient level of bothersomeness to be problematic at the behavioral and neural levels. Further studies to replicate this observation with a priori predictions regarding the specific ROIs that correlated with borthersomeness in our study will be an important next step. Whilst we interpret responses of “other” thoughts during the SART as non-ruminative (and our correlational data are consistent with this interpretation: “other” thoughts were correlated with fewer errors and slower RTs, whereas “unresolved problem” thoughts were correlated with more errors), due to the nature of the task design, we did not ask participants to report on the content of “other” thoughts and it is possible that some of these may have pertained to other problems or worries. Future studies that distinguish “other worries” from “other thoughts not related to ongoing problems or concerns” will help to further elucidate this distinction. We estimated a 12s epoch around problem responses on the thought probes to examine momentary shifts in rumination at the neural level. Given that we could not continuously assess thought content during these time windows, and it is possible that participants may have experienced some task-focus within these (e.g., a return to task-focused thought after a prompt). However, because we recruited participants high in trait rumination, who experience difficulties shifting away from ruminative thoughts, and for 77% of our sample the average number of consecutive problem responses on the thought probes was greater than 1, it seems likely that the probes were not reliably prompting a return to task-focused thoughts, but rather that most of our participants continued to experience problem-focused, ruminative thoughts. Were it the case that some participants experienced task-focused thoughts during these epochs, our analyzes would be more conservative as this should make it harder to detect any effect, and so it would not invalidate our findings. Due to experimenter error, 4 participants had relatively low (below 4) ratings of how much their ruminative problem bothers them now. Excluding these participants did not qualitatively alter the results, with the exception of a positive correlation between mean length of state rumination and RT variability emerging. Finally, the sample size for our study was determined by a power calculation for the primary outcome of the clinical trial within which the study was embedded (Langenecker et al., 2024; Roberts et al., 2021). Posthoc power calculations indicated that the study had good power to detect the observed behavioral findings within our sample. However, an a priori power analysis to determine the optimal sample size for the imaging analyzes would be desirable, particularly given the exploratory nature of some of these analyzes.

In summary, we captured the dynamic nature of idiographic state rumination, and modelled associations with IC-RS at both neural and behavioral levels in a sample of youth with elevated trait depressive rumination. We found that in high ruminating youth, uninstructed provoked state rumination is associated with impaired application of EFs, increased engagement of CCN and SEN regions during rumination, and increased DMN engagement during IC-RS failures. Moreover, we found increased engagement of DMPFC (and dorsal ACC) during state rumination, consistent with the hypothesis that DMPFC dysregulation in individuals at risk of recurrent depression may facilitate negative self-referential thought and impair EF.

## Supplementary Material

1

## Figures and Tables

**Table 1:** Sample characteristics and SART thought probe responses and performance indices.

	N	Minimum	Maximum	Mean	Std. Deviation
Age (years)	53	14	18	15.89	0.95
RRS total	53	30	80	58.60	11.64
Brooding	53	5	20	13.64	3.59
RADS-SF total	53	17	37	28.83	4.99
CDRS total	53	9	57	35.57	8.78
SCARED-GAD	53	0	18	11.43	4.49
Percent Rumination (PercentRum)	53	2.22	66.67	25.40	16.59
Percent Task	53	4.44	84.44	42.80	18.35
Percent Other	53	0.00	62.22	28.43	15.09
Percent Missing	53	0.00	40.00	3.37	7.90
Ave length rum (MeanDurRum)	53	1.00	7.25	1.60	0.97
Max length rum (MaxDurRum)	53	1.00	22.00	3.17	3.30
Errors of Commission (Comms)	53	0.00	63.00	21.25	16.84
Errors of Omission	53	2.00	143.00	41.11	43.19
RT Correct Go (ms)	53	174.40	766.75	527.30	128.97
SD of RT Correct Go	53	63.65	369.99	155.67	64.45

RRS total = Ruminative Response Scale total score, Brooding = RRS brooding subscale score, RADS-SF total = Reynolds Adolescent Depression Scale Short-Form total score, CDRS total = Children’s Depression Rating Scale – Revised total score, SCARED total = Screen for Child Anxiety Related Disorders total score, SCARED-GAD = SCARED generalized anxiety score.

**Table 2:** Coordinates and locations of effects for TP_UnresolvedProblem minus TP_Task and rumination measures.

Contrasts	Lobe/Region	BA	MNI Coordinates			Peak	Cluster K
			x	y	z	Z	mm3
Problem-Task	Frontal						
	Left-Frontal Eye Fields	8	−36	18	32	4.27	3208
	Left-DLPFC (dorsal)	9	−2	42	40	4.23	13344
	Left-PreMotor + Supplementary Motor	6	−40	12	48	3.64	1240
	Left-Broca-Triangle Parietal	45	−40	34	6	3.46	1560
	Left-Angular Gyrus Temporal	39	−50	−66	32	4.44	7672
	Right-Temporal pole Cerebellum	38	48	10	−28	3.83	976
	Right Cerebellum		40	−72	−36	3.75	1272
Task-Problem	Parietal						
	Left-Precuneus (Visuo Motor)	7	−18	−54	66	4.64	9392
	Right-Precuneus (Visuo Motor) Frontal	7	24	−48	70	3.73	1368
	Left-Insula	13	−40	2	10	4.57	1192
	Left-Ventral Anterior Cingulate Subcortical	24	−2	−4	48	4.11	688
	Right-Cerebellum		24	−52	−24	3.83	1160
RRS Positive activation	Parietal						
	Right-Angular Gyrus	39	52	−48	22	4.74	3600
	Left-Sensory Assoc	5	−14	−28	52	4.03	1144
	Precuneus (Left-Visuo Motor) Frontal	7	−18	−44	68	3.93	1056
	Right-Pre Motor + Supplementary Motor	6	2	−22	70	4.15	3360
	Left-Broca-Triangle	45	−42	34	8	4.15	744
	Left-Supramarginal Gyrus	40	−66	−30	22	4.12	3528
	Left-Dorsal PCC	31	−12	−44	42	3.88	984
	Right-Insula	13	38	−20	2	3.72	968
	Left-Anterior PFC	10	−26	46	−2	3.63	1624
	Left-Ventral Anterior Cingulate	24	−2	−6	46	3.51	864
	Right-Front Eye Fields	8	2	28	44	3.31	896
	Subcortical Cerebellum		36	−56	−38	4.36	4640
	Left-Caudate		−10	10	18	4.16	1432
Rum Percent positive activation	Parietal						
	Left-Supramarginal Gyrus	40	−66	−32	22	4.03	1528
	Right-Supramarginal Gyrus	40	66	−32	26	3.66	1328
Interaction RRS and percent rumination negative activation	Parietal						
	Left-Supramarginal Gyrus	40	−66	–30	22	4.01	880
	Right-Supramarginal Gyrus	40	66	–32	26	3.73	808
Interaction RRS and percent rumination positive activation	None						
RRS negative activation	None						
Percent rumination negative activation	None						

BA = Brodmann’s area; MNI coordinates = Montreal Neurologic Institute coordinates; DLPFC = Dorsolateral Prefrontal cortex; PCC = Posterior Cingulate Cortex; PFC = Prefrontal Cortex

## Footnotes

1 Posthoc power calculations indicated that for the correlation between state rumination and commission errors, with a sample size of 48 power was 0.940, and for the correlation with RTs to correct go trials, with a sample size of 48 power was 0.905. Power was therefore not a concern with respect to examining the effect of excluding these participants on our findings for the main hypotheses regarding the association between state rumination and SART performance.
