# Supplementary material for: State rumination predicts inhibitory control failures and dysregulation of default, salience, and cognitive control networks in youth at risk of depressive relapse: Findings from the RuMeChange trial
Source: J Affect Disord Rep. Author manuscript; Available in PMC 2024 May 20. (PMC11105748; doi:10.1016/j.jadr.2024.100729)
Supplement: 1 [file NIHMS1989651-supplement-1.docx]

| **Supplemental Table 1.** **Coordinates and locations of effects for Commissions minus Targets and Rumination Measures** | | | | | | | |
| --- | --- | --- | --- | --- | --- | --- | --- |
| **Contrast** | **Region** | **BA** | **MNI coordinates** | | | **Peak** | **Cluster** |
|  |  |  | x | y | z | Z | mm3 |
| **Commission-Targets** |  |  |  |  |  |  |  |
|  | Frontal |  |  |  |  |  |  |
|  | Right-Pars Orbitalis | 47 | 32 | 22 | -12 | 6.94 | 67040 |
|  | Left-Insula | 13 | -42 | 14 | 0 | 6.81 | 10296 |
|  | Left-dlPFC | 9 | -42 | 30 | 34 | 5.13 | 7024 |
|  | Left-Broca-Opercularis | 44 | -46 | 4 | 18 | 4.86 | 2432 |
|  | Left-PreMotor + Supplementary Motor | 6 | -16 | 8 | 62 | 4.81 | 1536 |
|  | Parietal |  |  |  |  |  |  |
|  | Right-Supramarginal Gyrus | 40 | 54 | -36 | 42 | 5.73 | 15632 |
|  | Left-Supramarginal Gyrus | 40 | -60 | -32 | 46 | 5.25 | 14312 |
|  | Right-Precuneus (Visuo-Motor) | 7 | 12 | -66 | 44 | 4.63 | 3336 |
| **Targets-Commission** |  |  |  |  |  |  |  |
|  | Frontal |  |  |  |  |  |  |
|  | Left-Primary Motor | 4 | -34 | -26 | 62 | 6.66 | 15656 |
|  | Right-Subgenual Anterior Cinguate | 25 | 4 | 10 | -10 | 6.18 | 3856 |
|  | Right-Anterior PFC | 10 | 4 | 54 | -6 | 6.17 | 5176 |
|  | Left-PreMotor + Supplementary Motor | 6 | -6 | -14 | 52 | 5.24 | 3552 |
|  | Left-Frontal Eye Fields | 8 | -20 | 30 | 40 | 4.78 | 6096 |
|  | Right-PreMotor + Supplementary Motor | 6 | 60 | 2 | 22 | 4.7 | 10848 |
|  | Right-Frontal Eye Fields | 8 | 24 | 30 | 46 | 4.66 | 1896 |
|  | Right-Pars Orbitalis | 47 | 36 | 36 | -14 | 4.35 | 616 |
|  | Left-Pars Orbitalis | 47 | -34 | 36 | -12 | 3.83 | 1136 |
|  | Left-Broca-Triangle | 45 | -56 | 24 | 14 | 3.82 | 912 |
|  | Parietal |  |  |  |  |  |  |
|  | Left-Ventral Posterior Cingulate | 23 | -8 | -50 | 10 | 6.73 | 71952 |
|  | Left-Angular Gyrus | 39 | -40 | -70 | 32 | 5.44 | 4760 |
|  | Left-Medial Temporal Gyrus | 21 | -60 | -8 | -16 | 5.16 | 4600 |
|  | Temporal |  |  |  |  |  |  |
|  | Right-Medial Temporal Gyrus | 21 | 56 | -2 | -20 | 5.65 | 9304 |
|  | Subcortical |  |  |  |  |  |  |
|  | Left-Putamen |  | -32 | -10 | 2 | 5.13 | 1576 |
| **RRS Positive** |  |  |  |  |  |  |  |
|  | Frontal |  |  |  |  |  |  |
|  | Left-Anterior PFC | 10 | -4 | 62 | 14 | 5.46 | 64848 |
|  | Left-PreMotor + Supplementary Motor | 6 | -48 | 2 | 12 | 5.29 | 3104 |
|  | Left-Ventral Anterior Cingulate | 24 | -2 | 34 | 2 | 5.07 | 10304 |
|  | Left-Pars Orbitalis | 47 | -34 | 38 | -10 | 4.97 | 3144 |
|  | Right-dlPFC | 9 | 12 | 38 | 32 | 4.58 | 2936 |
|  | Parietal |  |  |  |  |  |  |
|  | Left-Angular Gyrus | 39 | -36 | -76 | 30 | 4.63 | 1640 |
|  | Right-Angular Gyrus | 39 | 52 | -56 | 26 | 4.61 | 1520 |
|  | Right-Precuneus (VisuoMotor) | 7 | 32 | -82 | 38 | 4.53 | 2120 |
|  | Right-Primary Motor | 4 | 62 | -2 | 34 | 4.49 | 3736 |
|  | Right-Precuneus (VisuoMotor) | 7 | 34 | -68 | -18 | 4.43 | 728 |
|  | Right-Medial Temporal Gyrus | 21 | 56 | -32 | -6 | 4.42 | 896 |
|  | Left-Precuneus (VisuoMotor) | 7 | -32 | -68 | 54 | 4.24 | 960 |
|  | Temporal |  |  |  |  |  |  |
|  | Right-Superior Temporal Gyrus | 22 | 46 | 6 | -18 | 4.72 | 1096 |
|  | Right-Superior Temporal Gyrus | 22 | 46 | -34 | 18 | 4.51 | 1944 |
|  | Left-Medial Temporal Gyrus | 21 | -52 | -26 | -10 | 4.46 | 1368 |
|  | Occipital |  |  |  |  |  |  |
|  | Right-Visual Association | 19 | 28 | -50 | -2 | 4.77 | 1576 |
|  | Right-Secondary Visual | 18 | 30 | -90 | 18 | 4.22 | 1328 |
|  | Subcortical |  |  |  |  |  |  |
|  | Right-Optic tract |  | 8 | 4 | -16 | 4.81 | 832 |
|  | Left-Caudate |  | -8 | 8 | 22 | 4.38 | 752 |
| **RRS Negative** | Frontal |  |  |  |  |  |  |
|  | Right-dlPFC (dorsal) | 9 | 48 | 42 | 24 | 5.18 | 7768 |
|  | Right-dlPFC (lateral) | 46 | 56 | 36 | 10 | 5.18 | 1592 |
|  | Left-Frontal Eye Fields | 8 | -4 | 22 | 56 | 4.66 | 1168 |
|  | Right-dlPFC (dorsal) | 9 | 10 | 46 | 30 | 4.49 | 824 |
|  | Right-Frontal Eye Fields | 8 | 38 | 10 | 34 | 4.36 | 1800 |
|  | Right-PreMotor + Supplementary Motor | 6 | 20 | 6 | 58 | 4.3 | 1904 |
|  | Left-Broca-Triangle | 45 | -56 | 24 | 4 | 4.09 | 1096 |
|  | Parietal |  |  |  |  |  |  |
|  | Right-Supramarginal Gyrus | 40 | 62 | -36 | 44 | 4.73 | 2480 |
|  | Right-Medial Temporal Gyrus | 21 | 54 | -16 | -14 | 4.67 | 872 |
|  | Right-Dorsal Posterior Cingulate Cortex | 31 | 8 | -48 | 46 | 4.4 | 2328 |
|  | Right-Ventral Posterior Cingulate | 23 | 24 | -48 | 24 | 4.39 | 1240 |
|  | Left-Angular Gyrus | 39 | -64 | -42 | 26 | 4.35 | 944 |
|  | Left-Precuneus (VisuoMotor) | 7 | -18 | -62 | 32 | 4.05 | 808 |
|  | Temporal |  |  |  |  |  |  |
|  | Right-Fusiform | 37 | 54 | -48 | 0 | 3.87 | 2024 |
|  | Occipital |  |  |  |  |  |  |
|  | Right-Primary Visual | 17 | 2 | -94 | 4 | 3.64 | 816 |
|  | Subcortical |  |  |  |  |  |  |
|  | Right-Caudate |  | 16 | 10 | 18 | 5.03 | 36416 |
|  | Right-Caudate |  | 22 | -24 | 18 | 4.52 | 1176 |
|  | Right Cerebellum |  | 28 | -66 | -24 | 4.45 | 904 |
|  | Right Cerebellum |  | 4 | -48 | -28 | 4.35 | 3560 |
| **Positive Interaction, RRS and Percent Rumination** | Frontal |  |  |  |  |  |  |
|  | Left Pars-Orbitalis | 47 | -32 | 40 | -10 | 4.7 | 1048 |
|  | Left Primary Motor Cortex | 4 | -28 | -30 | 54 | 4.54 | 3056 |
|  | Parietal |  |  |  |  |  |  |
|  | Right Sensory Motor Cortex | 5 | 22 | -38 | 54 | 4.93 | 6016 |
|  | Right-Dorsal Posterior Cingulate Cortex | 31 | -2 | -64 | 16 | 4.64 | 792 |
| **Negative Interaction, RRS and Percent Rumination** | Frontal |  |  |  |  |  |  |
|  | Right-dlPFC (dorsal) | 9 | 46 | 44 | 28 | 5.46 | 3512 |
|  | Right-dlPFC (lateral) | 46 | 56 | 36 | 10 | 5.14 | 872 |
|  | Right-dlPFC (dorsal) | 9 | 36 | 24 | 26 | 4.69 | 1312 |
|  | Right-Broca-Opercularis | 44 | 34 | 14 | 14 | 4.64 | 2384 |
|  | Right-Ventral Anterior Cingulate | 24 | 2 | 12 | 30 | 4.54 | 1448 |
|  | Right-Pars Orbitalis | 47 | 50 | 20 | -14 | 4.31 | 1208 |
|  | Right-PreMotor + Supplementary Motor | 6 | 52 | 6 | 32 | 4.15 | 920 |
|  | Parietal |  |  |  |  |  |  |
|  | Right-Supramarginal Gyrus | 40 | 62 | -38 | 42 | 5.11 | 1192 |
|  | Temporal |  |  |  |  |  |  |
|  | Left-Medial Temporal Gyrus | 21 | -60 | -36 | 4 | 4.89 | 2096 |
|  | Subcortical |  |  |  |  |  |  |
|  | Right Putamen |  | 22 | 4 | 8 | 4.32 | 896 |
|  | Left Putamen |  | -14 | 12 | 2 | 4.27 | 1400 |
